# First Trimester Maternal Vitamin D Status and Risks of Preterm Birth and Small-For-Gestational Age

**DOI:** 10.3390/nu11123042

**Published:** 2019-12-13

**Authors:** Isabelle Monier, Amandine Baptiste, Vassilis Tsatsaris, Marie-Victoire Senat, Jacques Jani, Jean-Marie Jouannic, Norbert Winer, Caroline Elie, Jean-Claude Souberbielle, Jennifer Zeitlin, Alexandra Benachi

**Affiliations:** 1 Université de Paris, CRESS, INSERM, INRA, F-75004 Paris, France; jennifer.zeitlin@inserm.fr; 2 Department of Obstetrics and Gynaecology, Antoine Béclère Hospital, AP-HP, University Paris Saclay, F-92140 Clamart, France; alexandra.benachi@aphp.fr; 3 URC/CIC Paris Descartes Necker Cochin, Necker-Enfants Malades Hospital, AP-HP, F-75015 Paris, France; 4 Department of Obstetrics, Cochin Hospital, AP-HP, Paris-Descartes University, F-75014 Paris, France; 5 Department of Obstetrics and Gynecology, Bicêtre Hospital, AP-HP, Université Paris Saclay, F-94270 Kremlin Bicêtre, France; 6 Department of Obstetrics and Gynecology, University Hospital Brugmann, Université Libre de Bruxelles, B-1000 Brussels, Belgium; 7 Fetal Medecine Department, Armand Trousseau Hospital, AP-HP, UPMC-Sorbonne Université, F-75012 Paris, France; 8 Department of Obstetrics and Gynecology, University Hospital of Nantes, CIC Mere enfant Nantes, NUN, INRA, UMR 1280, Phan, Nantes University, F-44000 Nantes, France; 9 Laboratoire d’Explorations Fonctionnelles, Necker-Enfants malades Hospital, AP-HP, F-75015 Paris, France

**Keywords:** vitamin D, 25-hydroxyvitamin D, pregnancy, preterm birth, small-for-gestational age, skin color

## Abstract

Maternal 25-hydroxyvitamin D (25-OHD) deficiency during pregnancy may increase the risk of preterm and small-for-gestational age (SGA) birth, but studies report conflicting results. We used a multicenter prospective cohort of 2813 pregnant women assessed for 25-OHD levels in the first trimester of pregnancy to investigate the association between maternal 25-OHD concentrations and risks of preterm birth (<37 weeks) and SGA (birthweight <10th percentile). Odds ratios were adjusted (aOR) for potential cofounders overall and among women with light and dark skin separately, based on the Fitzpatrick scale. 25-OHD concentrations were <20 ng/mL for 45.1% of the cohort. A total of 6.7% of women had a preterm birth. The aOR for preterm birth associated with the 1st quartile of 25-OHD concentrations compared to the 4th quartile was 1.53 (95% confidence interval (CI): 0.97–2.43). In stratified analyses, an association was observed for women with darker skin (aOR = 2.89 (95% CI: 1.02–8.18)), and no association with lighter skin. A total of 11.9% of births were SGA and there was no association overall or by skin color. Our results do not provide support for an association between maternal first trimester 25-OHD deficiency and risk of preterm or SGA birth overall; the association with preterm birth risk among women with darker skin requires further investigation.

## 1. Introduction

Vitamin D is generated in the liver and then in the kidneys and is commonly assessed by the measurement of 25-hydroxyvitamin D (25-OHD) levels [1]. Vitamin D has an important role in bone and mineral metabolism and was historically used for rickets prevention. During pregnancy, vitamin D may play a role in the prevention of infections and fetal bone growth [2,3,4]. Therefore, vitamin D supplementation for pregnant women has been seen as a potential intervention to reduce the risk of preterm birth and small-for-gestational age (SGA) that are main causes of perinatal morbidity and mortality [5,6,7]. One hypothesis focuses on the role of maternal vitamin D in placenta inflammation and the development of bacterial vaginosis, which are known to be associated with the risk of preterm birth [2,3]. Vitamin D may also have an effect on fetal bone development and fetal growth. One study found that the expression of placenta vitamin D receptor was decreased among pregnancies with fetuses with growth restriction [8].

Some research has provided evidence for the existence of an association between vitamin D and preterm and SGA birth, but results have not been consistent. Meta-analyses of randomized controlled trials have reported an association between lower maternal vitamin D concentrations and an increased risk of preterm birth and low birthweight <2500 g [9], a higher risk of SGA but not preterm birth [10], and no association with either outcome [11]. Some observational studies found that vitamin D deficiency increased the risk of preterm birth [12,13] or SGA [14,15,16,17], whereas others have not [18,19,20,21]. Many of these studies have methodological limitations, however. Vitamin D status in the first trimester is of the most interest for prevention, but vitamin D is often measured later in pregnancy [14,16,22,23,24]. Further, most observational studies have been single-centre studies [12,14,15,18,19,20] or were not designed for the assessment of maternal vitamin D status during pregnancy [12,14,15,19,22]. Several studies also found that the relationship between maternal vitamin D concentrations and the risk of preterm and SGA birth differed for white and black women, based on race and ethnicity used as a proxy for skin color [13,14,15,25]. Skin color is one factor that is highly related to vitamin D absorption, with a higher risk of vitamin D deficiency in dark-skinned women [1,26], but this information has rarely been available in these research studies [9].

In this study, our aim was to investigate the association between maternal first trimester vitamin D concentrations and the risks of preterm and SGA birth using a large prospective cohort designed for the assessment of maternal vitamin D during pregnancy which includes 25-OHD concentrations measured between 10 and 14 weeks of gestation as well as data on skin color.

## 2. Materials and Methods

### 2.1. Study Design

The FEPED study is an observational prospective cohort conducted from April 2012 through July 2014 in five French and one Belgium maternity units [27,28]. The primary aim of this study was to assess the prevalence of vitamin D deficiency over the course of pregnancy and the association between vitamin D concentrations and preeclampsia [28]. Eligible women were aged over 18 years old with a singleton pregnancy between 10 and 14 weeks of gestational age at enrolment. Enrolment took place at the first prenatal visit in the maternity units participating in the study. Women were not eligible if they met one of the following criteria: hypercalcemia >2.65 mmol/L or other known phosphocalcic diseases, hypertension at the beginning of the pregnancy (defined as a systolic blood pressure >140 mmHg or a diastolic blood pressure >90 mmHg), renal insufficiency (creatinine concentration >120 μmol/L), bone disease, Lithium treatment, bowel malabsorption or kidney stone disease. Each woman received information and provided written consent to participate in the study. The study was approved by the National Data Protection Authority (CNIL no. 911432), and the committee for the protection of people participating in biomedical research (2011/13 NICB; 31/05/2011).

At enrolment, a physician completed a questionnaire to ensure that women met the inclusion criteria. In this questionnaire, skin color was assessed using the Fitzpatrick scale [29]. The Fitzpatrick classification includes six skin types according to phenotype (skin, hair and eyes color) and skin tolerance to ultraviolet radiation exposure, based on a brief interview with the women. Type I indicates pale skin and type VI very dark skin. Women were subsequently grouped into those with lighter skin (skin type I through IV) and those with darker skin (skin type from V to VI), in line with previous investigations [26,28].

After delivery, sociodemographic and clinical data were abstracted from medical records by personnel trained for the study. Sociodemographic maternal characteristics included parity, maternal age, height, prepregnancy weight, ethnicity and smoking status before and during pregnancy. Information was also collected on medical and obstetrical history (diabetes, chronic hypertension, auto-immune diseases, obstetrical history of SGA infant, preterm birth, stillbirth and pre-eclampsia), complications of the current pregnancy (pre-eclampsia, HELLP syndrome, gestational diabetes), the delivery and the infant’s health status at birth.

### 2.2. Study Population

**Figure 1:**
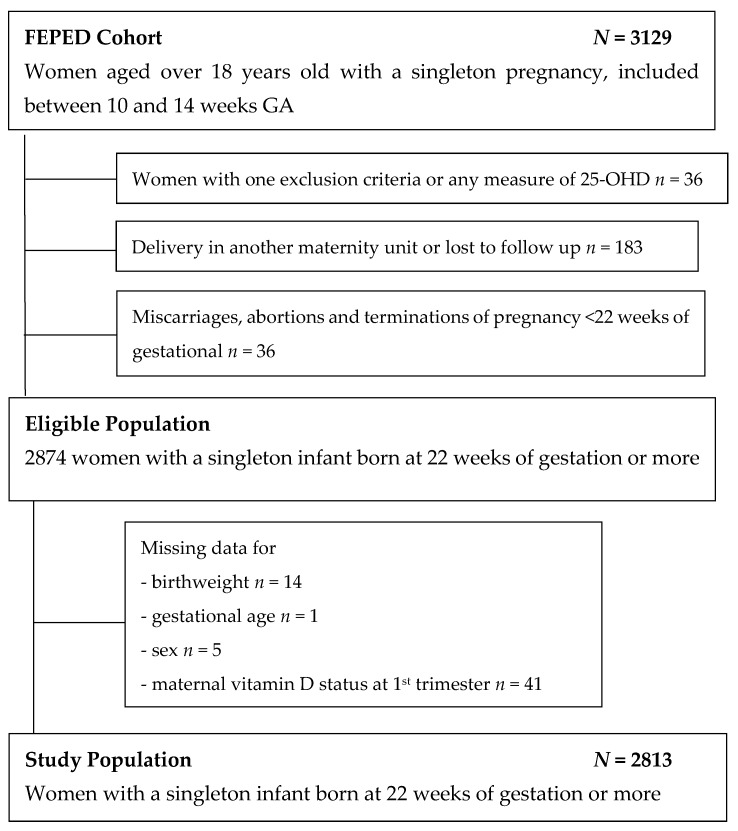
Flow chart.

In the original cohort, 3129 women were included; 36 women were subsequently excluded because they were discovered to have an exclusion criteria or had no blood test to measure 25-OHD concentrations. We further excluded women who delivered outside of participating maternity units or were lost to follow-up (*n* = 183), miscarriages, abortions and terminations of pregnancy before 22 weeks of gestation (*n* = 36). Cases with missing data for birthweight (*n* = 14), gestational age (*n* = 1), infant sex (*n* = 5) and 25-OHD maternal concentrations in the first trimester of pregnancy (*n* = 41) were also excluded. The study population included 2813 women. (Figure 1)

### 2.3. Definition of Outcomes

Preterm birth was defined as a delivery <37 weeks of gestation and SGA as a birthweight <10th percentile according to intrauterine growth reference adapted to the French population [30]. We selected these outcomes, as opposed to gestational age or birthweight, as continuous variables, because they have been used in most studies reported in systematic reviews [9,10,11]. In France, the first routine ultrasound is recommended between 11 + 0 and 13 + 6 weeks of gestation for pregnancy dating, based on the crown–rump length (CRL), in accordance with the date of the last menstrual period (LMP). In case of disagreement between CRL and the LMP, CRL is used for pregnancy dating [31].

### 2.4. Maternal 25-OHD Serum

All women participating in the study provided blood samples between 10 and 14 weeks of gestation to measure their 25-OHD concentration, which is the best assessment of vitamin D levels in the human body [1]. Information was recorded on gestational age and season at blood draw. All blood samples were centrifuged and stored locally at −20 °C and transferred monthly to the department of Physiology of Necker University hospital (Paris, France). Serum 25-OHD levels were measured by radioimmunoassay (RIA) after pretreatment with acetonitrile (Diasorin, Stillwater, MN, USA) [32]. The limit of quantification determined in our laboratory was 4 ng/mL and the inter-assay coefficient of variation was <10%. Quality control was ensured through the participation of this laboratory in the DEQAS quality assurance program.

Despite recommendations on the prevention of vitamin D deficiency from the Institute of Medicine and the US Endocrine Society [33,34], there is no consensus on the definition of vitamin D deficiency [35]. We choose to assess maternal 25-OHD concentrations using two methods: (1) using clinical cut-offs based on the US Endocrine Society recommendations [33] (<20, 20–29, 30+ ng/mL) and (2) in quartiles of the observed distribution. In France and in Belgium, women are not routinely supplemented with vitamin D before pregnancy [36]. 

### 2.5. Statistical Analysis

We described the maternal characteristics of the study population and estimated means and standard deviations (SD) of maternal 25-OHD concentrations in the first trimester overall and by maternal characteristics. We then calculated the proportion of women with 25-OHD concentrations <20 ng/mL in the first trimester of pregnancy by selected characteristics. Based on the scientific literature and biological plausibility, we selected co-variables as possible confounders of the association between vitamin D concentrations and preterm birth and SGA, including maternal age, parity, pre-pregnancy body mass index (BMI), smoking, medical and obstetrical history. We also included season at blood draw, skin color and ethnicity, since these were hypothesized to be strongly related to vitamin D status and there is increasing evidence of an association with perinatal outcome. Seasonal variations in respiratory and viral infections, as well as in the temperature, impact on preterm birth [37,38,39,40], while skin color is related to ethnicity, which has an independent effect on preterm birth and SGA; recent research suggests that skin tone may also be directly relevant to the investigation of perinatal outcomes [41,42,43].

Logistic regression was also used to estimate adjusted odds ratios (aOR) for preterm and SGA birth for the groups, defined by quartile and clinical cutoffs. Based on the hypothesis that there may be an interaction between our outcomes and skin color, we carried out analyses stratified by skin color according to the Fitzpatrick scale [29]. As a sensitivity analysis, models were run with gestational age and birthweight as continuous variables. Finally, in order to assess whether our cut-off points adequately described risk patterns, we used restricted cubic splines with four knots to represent the association between 25-OHD maternal concentrations and the risk of preterm and SGA birth in the overall sample and by skin color separately. Analyses were performed using Stata 13.0 software (StataCorp LP, College Station, TX, USA).

## 3. Results

### 3.1. Maternal Characteristics of the Study Population

Table 1 presents the maternal characteristics of the study population and the means of maternal 25-OHD concentrations in the first trimester of pregnancy. Mean 25-OHD concentration was 22.2 ± 10.3 ng/mL and differed by maternal characteristics, as well as season of blood draw. Women with light skin (type I to IV) had mean 25-OHD concentrations of 23.1 ± 10.2 ng/mL compared to 18.9 ± 10.1 ng/mL for women with dark skin (type V and VI).

### 3.2. Factors Related to Low Maternal 25-OHD Levels

Overall, 45.1% of women had low 25-OHD levels <20 ng/mL in the first trimester of pregnancy. There was a higher proportion of women with low 25-OHD levels among younger women (56.8%), and those with a BMI before pregnancy of ≥30 kg/m^2^ (51.8%), Africans and those with a dark skin color (60.4%) (Table 2). In contrast, the proportion of women with 25-OHD levels <20 ng/mL was lower when blood draw in the first trimester of pregnancy was tested in summer (26.3%) and in autumn (40.7%).

### 3.3. Association between Maternal 25-OHD Concentrations in the First Trimester of Pregnancy and Risks of Preterm and Small-for-Gestational Age Birth

Twenty-seven percent of women had 25-OHD concentrations <15 ng/mL and 45.1% had concentrations <20 ng/mL. (Table 3) There was no difference in the means of gestational age at blood draw between preterm and term birth (12.68 ± 0.80 vs. 12.77 ± 0.81, *p* = 0.144), and SGA and non-SGA birth (12.73 ± 0.76 vs. 12.77 ± 0.81, *p* = 0.391) (results not shown). Compared to the 4th quartile (30 ng/mL^+^), the adjusted odds ratio associated with 25-OHD concentrations in the 1st quartile was 1.53 (95% CI: 0.97–2.43)) for the risk of preterm birth and 1.07 (95% CI: 0.75–1.54) for the risk of having an SGA infant.

The interaction between skin color and vitamin D concentrations was significant for the risk of preterm birth (*p* value = 0.050) but not for SGA (*p* value = 0.708). Analyses stratified by skin color showed that women with light skin had no excess risk of preterm birth, whereas the risk of preterm birth for women with dark skin (type V to VI) was increased for the first quartile of 25-OHD (aOR = 2.89 (95% CI: 1.02–8.18)). Results were similar using clinical cut-offs, with an aOR of 2.63 (95% CI: 0.95–7.27) for 25-OHD concentrations <20 ng/mL compared to those with 25-OH levels >30 ng/mL. No association was observed for SGA using quartiles or clinical cut-offs. Analyses with birthweight and gestational age as continuous variables confirmed the absence of an association between low 25-OHD levels with birthweight in the overall sample and an interaction with skin colour for gestational age (*p* = 0.05). (Table S1)

### 3.4. Association between 25-OHD Maternal Concentrations and Risks of Preterm and SGA Birth Using Restricted Cubic Splines

**Figure 2:**
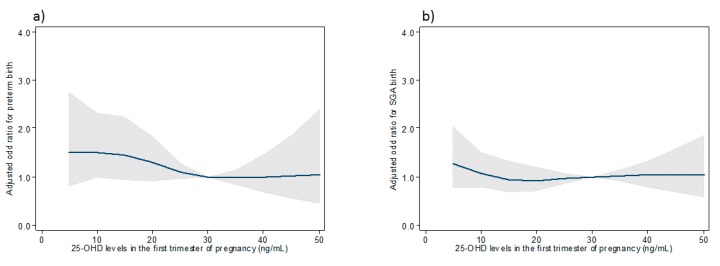
Association between 25-OHD concentrations in the first trimester of pregnancy and the risk of preterm (**a**) and SGA birth (**b**) in the overall sample.

**Figure 3:**
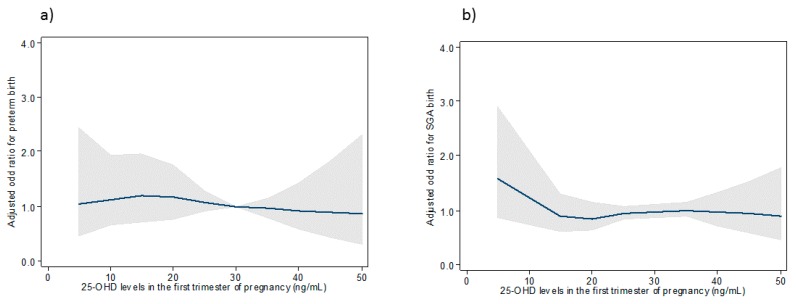
Association between 25-OHD concentrations in the first trimester of pregnancy and the risk of preterm (**a**) and SGA birth (**b**) for women with light skin (type I to type IV).

**Figure 4:**
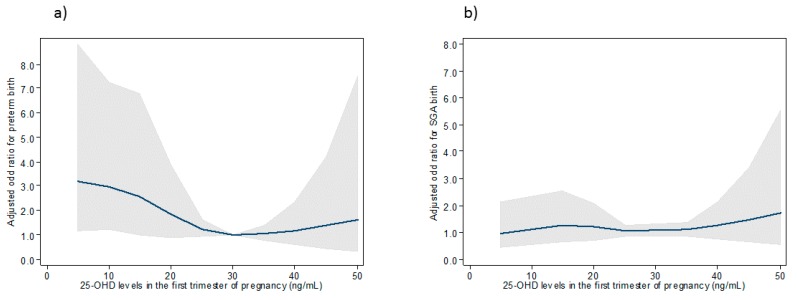
Association between 25-OHD concentrations in the first trimester of pregnancy and the risk of preterm (**a**) and SGA birth (**b**) for women with dark skin (type V to type VI).

Restricted cubic splines were used to investigate the association between maternal 25-OHD concentrations and the risk of preterm and SGA birth adjusted for maternal age, parity, BMI before pregnancy, medical or obstetrical history, smoking, ethnicity, skin color and season at blood draw overall. Figures show that the risk of preterm birth and having an SGA infant were not different, regardless of maternal vitamin D concentrations in the overall sample and for women with light skin color. (Figure 2 and Figure 3) For women with dark skin color, the risk of preterm birth was elevated for the lowest maternal 25-OHD levels and no association was found with the risk of SGA birth (Figure 4).

## 4. Discussion

In our cohort, almost half of pregnant women had 25-OHD concentrations in the first trimester of pregnancy below the lower clinical cut-off point of <20 ng/mL, one-quarter had 25-OHD concentrations of <15 ng/mL, while only 24% of women had 25-OHD levels above 30 ng/mL. No association was found between low maternal vitamin D concentrations and the risk of preterm and SGA birth in the overall sample and among women with a lighter skin. In contrast, an increased risk of preterm birth, but not SGA, was observed for women with darker skin.

Our study has several strengths. We used data from a large multicentre prospective cohort explicitly designed to investigate the association between maternal vitamin D concentrations and perinatal outcomes [27,28]. All pregnant women had a blood draw between 10 and 14 weeks of gestation, as opposed to other studies that used wide gestational age limits for the assessment of 25-ODH concentrations [12,13,14]. France and Belgium have a temperate climate and variability in sun exposure was also limited because participating centres were at similar latitudes [27]. Also, in contrast to many previous analyses, we had standardised measures of skin color. Limitations include the observational design, which means it is not possible to demonstrate a causal relationship. We could not consider the potential for residual confounding remains and, notably, the mother’s socioeconomic characteristics. However, smoking, prepregnancy BMI and ethnicity are related to socioeconomic position [44] and we included them in our models. Finally, the size of our sample was too small to investigate preterm birth subtypes (spontaneous or induced preterm birth).

Our aim was to assess the benefit of vitamin D supplementation in early pregnancy on perinatal outcomes and, thus, we did not examine the changes in vitamin D levels between the first and the third trimester of pregnancy. This change was examined in a previous analysis using the FEPED cohort. The increase of 25-OHD levels was estimated to 6.3 ng/mL (SD = 12.6) and 8.5 (SD = 11.5) in women with pre-eclampsia and in those with no pre-eclampsia [28]. In our study, we found that almost half of women had vitamin D deficiency (<20 ng/mL) in their first trimester of pregnancy; our study adds to limited research showing that vitamin D deficiency is highly prevalent among pregnant women [25,26,45,46]. A Belgian survey estimated that 82% of pregnant women had a vitamin D concentration <30 ng/mL in their first trimester of pregnancy, a little higher than the 75% in our study [47].

There was a higher proportion of women with low 25-OHD levels <20 ng/mL in the first trimester of pregnancy among obese women, Africans, darker skin colored and based on season at blood draw. Vitamin D absorption depends on body fat, skin pigmentation and sun exposure and these characteristics are thus well-known risks factors for vitamin D deficiency [1,26,48].

We investigated two pregnancy outcomes previously associated with vitamin D concentrations and for which plausible biological mechanisms have been suggested. Vitamin D could play a role in the regulation of genes involved in trophoblast invasion and in the angiogenesis for placenta implantation that could impact on fetal growth. However, our results related to SGA do not support an association with maternal vitamin D levels, corroborating a number of previous studies [11,23] but contrasting with others, including the results of the Cochrane Collaboration [5,9,49]. Our results also go against two studies showing an association with SGA among white, but not black women [14,15]. In addition, an increase in the production of inflammatory cytokines has been suggested in pregnant women with low vitamin D levels and this could increase the risk of preterm birth. Vitamin D is also involved in the immune system and low maternal vitamin D levels could be associated with bacterial vaginosis, which is a risk factor of preterm birth [50]. In our study, we found an elevated, but not significant effect in the overall population that reflected a high odds ratio, over two, among women with darker skin, and no association among women with lighter skin. This result similarly conflicts with multiple studies, including several meta-analyses of observational studies of preterm birth that have found an impact in general samples including women with light and dark skin [5,51]. However, our results are concordant with a previous study from the United States stratifying on race/ethnicity that showed the protective effects of vitamin D for spontaneous preterm births among black women, but not white women [13].

Our results, taken together with the body of conflicting literature on vitamin D and pregnancy outcomes, including the high study heterogeneity in meta-analyses, suggests that the association of vitamin D concentrations and these pregnancy outcomes may depend on the populations studied [5,9,10,11,49]. The differences between populations could be the result of residual confounding, as maternal vitamin D status depends on many factors, including diet and nutrition, vitamin supplementation, physical activity, socioeconomic status and race, all of which are independently related to pregnancy outcome. This means that some factors were inadequately measured and led to a spurious association [52]. However, population variation in sensitivity to vitamin deficiency may also explain discordant results, perhaps due to differences in the aetiology of preterm and SGA birth across populations. In the study showing an effect of vitamin D deficiency on risk of spontaneous preterm births among black women, the authors note that preterm deliveries occurring among black women have been found to be more often associated with infection [13]. In contrast, black women could be less sensitive to vitamin D deficiency, due to renal and skeletal adaptation [53], which was put forth as an explanation for the absence of association between a low vitamin D level and risk of SGA in black women [14,15]. Finally, the effects of vitamin D on birth outcomes may be different according to the timing of vitamin D assessment. In the meta-analysis of Chen et al., a positive association was found between maternal vitamin D deficiency and the risk of having an SGA infant (OR = 1.59 (95% CI: 1.14–2.22)) [49]. However, this association was no longer significant in the subgroup analysis of studies that measured vitamin D strictly during the first trimester of pregnancy (OR = 1.29 (95% CI: 0.95–1.74)). Further research, to assess biological pathways in light of these questions, is needed to design more informative interventional and observational studies.

In conclusion, our study does not provide support for a common biological pathway linking vitamin D concentrations in the first trimester of pregnancy to risks of preterm or SGA birth. However, further investigation is needed on potential differences in preterm birth risks among women with darker skin color.

## Figures and Tables

**Table 1:** Maternal characteristics and vitamin D concentration at the first trimester of pregnancy.

	All Women		
	*n* (%)	Mean 25-OHD ± SD (ng/mL)	*p*-Value
Total	2813	22.2 ± 10.3	
Maternal Age (years)			
18–24	257 (9.1)	19.4 ± 9.8	<0.001
25–34	1845 (65.6)	22.3 ± 10.2	
35+	711 (25.3)	22.9 ± 10.6	
BMI (kg/m^2^)			
<18.5	165 (5.9)	21.4 ± 10.9	0.001
18.5–24.9	1826 (65.1)	22.7 ± 10.3	
25–29.9	542 (19.3)	21.2 ± 10.3	
30+	270 (9.6)	20.9 ± 9.8	
Parity			
Nulliparous	1339 (47.6)	22.7 ± 10.4	0.008
Multiparous	1474 (52.4)	21.7 ± 10.2	
Smoking During Pregnancy			
Yes	329 (11.8)	22.6 ± 9.8	0.416
No	2447 (88.2)	22.1 ± 10.4	
Origin			
France	1567 (55.8)	23.8 ± 9.9	<0.001
Other European Countries	259 (9.2)	25.1 ± 10.1	
North African Countries	463 (16.5)	17.8 ± 10.1	
Other African Countries	299 (10.7)	19.1 ± 10.1	
Other Countries	218 (7.8)	20.3 ± 10.2	
Skin Color ^a^			
Light Skin (Type I to IV)	2197 (78.1)	23.1 ± 10.2	<0.001
Dark Skin (Type V to VI)	616 (21.9)	18.9 ± 10.1	
Season at Blood Draw ^b^			
Spring	726 (25.8)	19.9 ± 10.5	<0.001
Summer	715 (25.4)	26.3 ± 9.9	
Autumn	752 (26.7)	23.0 ± 10.0	
Winter	620 (22.0)	18.9 ± 9.2	
Medical or Obstetrical History ^c^			
Yes	458 (16.3)	21.4 ± 10.2	0.091
No	2355 (83.7)	22.3 ± 10.4	

^a^ Based on the Fitzpatrick scale. ^b^ Seasons were defined as follows: spring runs from 20 March to 19 June, summer from 20 June to 21 September, fall (autumn) from 22 September to 21 December, and winter from 22 December to 19 March. ^c^ Included diabetes, chronic hypertension, auto-immune diseases, obstetrical history of SGA infant, preterm birth, stillbirth and pre-eclampsia.

**Table 2:** Socio-demographic, medical and blood draw characteristics related to low maternal 25-OHD concentrations <20 ng/mL in the first trimester of pregnancy.

	Total	Low Maternal 25-OHD Levels <20 ng/mL	
	*N*	*n*/*N* (%)	Unadjusted OR (95% CI)
Total	2813	1268/2813 (45.1)	
Maternal Age (years)			
18–24	257	146/257 (56.8)	1.73 (1.29–2.30)
25–34	1845	815/1845 (44.2)	1.04 (0.87–1.23)
35+	711	307/711 (43.2)	Reference
BMI (kg/m^2^)			
<18.5	165	81/165 (49.1)	1.29 (0.93–1.77)
18.5–24.9	1826	781/1826 (42.8)	Reference
25–29.9	542	259/542 (47.8)	1.22 (1.01–1.48)
30+	270	140/270 (51.8)	1.44 (1.11–1.86)
Parity			
Nulliparous	1339	567/1339 (42.3)	0.80 (0.69–0.94)
Multiparous	1474	701/1474 (47.6)	Reference
Smoking during Pregnancy			
Yes	329	137/329 (41.6)	0.85 (0.68–1.08)
No	2447	1110/2447 (45.4)	Reference
Origin			
France	1567	589/1567 (37.6)	Reference
Other European Countries	259	83/259 (32.0)	0.78 (0.59–1.03)
North African Countries	463	295/463 (63.7)	2.91 (2.35–3.61)
Other African Countries	299	178/299 (59.5)	2.44 (1.89–3.14)
Other Countries	218	119/218 (54.6)	1.99 (1.50–2.65)
Skin Color ^a^			
Light Skin (Type I to IV)	2197	896/2197 (40.8)	Reference
Dark Skin (Type V to VI)	616	372/616 (60.4)	2.21 (1.84–2.65)
Season at Blood Draw ^b^			
Spring	726	404/726 (55.6)	Reference
Summer	715	188/715 (26.3)	0.28 (0.22–0.35)
Autumn	752	306/752 (40.7)	0.54 (0.44–0.67)
Winter	620	370/620 (59.7)	1.17 (0.94–1.46)
Medical or Obstetrical History ^c^			
Yes	458	224/458 (48.9)	1.20 (0.98–1.46)
No	2355	1044/2355 (44.3)	Reference

^a^ Based on the Fitzpatrick scale. ^b^ Seasons were defined as follows: spring runs from 20 March to 19 June, summer from 20 June to 21 September, fall (autumn) from 22 September to 21 December, and winter from 22 December to 19 March. ^c^ Included diabetes, chronic hypertension, auto-immune diseases, obstetrical history of SGA infant, preterm birth, stillbirth and pre-eclampsia.

**Table 3:** Vitamin D status in the first trimester of pregnancy and risk of preterm and small-for-gestational age status at birth in the overall sample and by skin color.

25-OHD Concentrations in the 1st Trimester of Pregnancy		Gestational Age at Birth				Birthweight			
	Total	<37 Weeks	≥37 Weeks	Crude OR (CI 95%)	Adjusted OR (CI 95%)	<10th Percentile	≥10th Percentile	Crude OR (CI 95%)	Adjusted OR (CI 95%)
	%	%	%			%	%		
Overall sample n (%)		189 (6.7)	2624 (93.3)			336 (11.9)	2477 (88.1)		
Level Cut-Off (ng/mL)									
<20	45.1	53.5	44.5	1.41 (0.96–2.07)	1.46 (0.96–2.22) ^a^	46.4	44.9	1.07 (0.80–1.43)	1.10 (0.80–1.52) ^a^
20–29	30.9	25.9	31.2	0.97 (0.63–1.50)	1.04 (0.67–1.63) ^a^	30.4	30.9	1.02 (0.74–1.39)	1.09 (0.79–1.51) ^a^
30+	24.1	20.6	24.3	Reference	Reference	23.2	24.2	Reference	Reference
In Quartiles (ng/mL)									
Q1: <15	27.0	34.4	26.6	1.52 (1.01–2.30)	1.53 (0.97–2.43) ^a^	27.4	27.0	1.05 (0.76–1.45)	1.07 (0.75–1.54) ^a^
Q2: 15–21	24.1	25.4	24.0	1.24 (0.80–1.92)	1.37 (0.86–2.17) ^a^	22.9	24.3	0.98 (0.70–1.37)	1.01 (0.71–1.44) ^a^
Q3: 22–29	24.7	19.6	25.1	0.91 (0.57–1.45)	0.99 (0.61–1.58) ^a^	26.5	24.5	1.12 (0.81–1.55)	1.18 (0.84–1.65) ^a^
Q4: 30+	24.1	20.6	24.3	Reference	Reference	23.2	24.2	Reference	Reference
									
Light Skin (Type I to IV) n (%)		131 (6.0)	2066 (94.0)			241 (11.0)	1956 (89.0)		
Level Cut-Off (ng/mL)									
<20	40.8	44.3	40.6	1.10 (0.71–1.71)	1.19 (0.73–1.93) ^b^	40.2	40.8	0.97 (0.69–1.36)	1.12 (0.78–1.62) ^b^
20–29	32.9	29.8	33.1	0.91 (0.57–1.47)	0.97 (0.60–1.58) ^b^	33.2	32.8	1.00 (0.70–1.42)	1.08 (0.75–1.54) ^b^
30+	26.3	25.9	26.3	Reference	Reference	26.6	26.3	Reference	Reference
In Quartiles (ng/mL)									
Q1: <15	23.2	25.9	23.0	1.14 (0.70–1.87)	1.15 (0.66–2.03) ^b^	23.6	23.1	1.01 (0.69–1.48)	1.22 (0.80–1.87) ^b^
Q2: 15–21	23.8	25.2	23.8	1.07 (0.65–1.76)	1.22 (0.72–2.05) ^b^	20.3	24.3	0.83 (0.56–1.22)	0.91 (0.60–1.37) ^b^
Q3: 22–29	26.6	22.8	26.9	0.86 (0.52–1.43)	0.93 (0.55–1.55) ^b^	29.5	26.3	1.11 (0.77–1.59)	1.18 (0.81–1.70) ^b^
Q4: 30+	26.3	25.9	26.3	Reference	Reference	26.6	26.3	Reference	Reference
									
Dark Skin (Type V to VI) n (%)		58 (9.4)	558 (90.6)			95 (15.4)	521 (84.6)		
Level cut-off (ng/mL)									
<20	60.4	74.1	59.0	2.43 (0.93–6.31)	2.63 (0.95–7.27) ^b^	62.1	60.1	1.13 (0.60–2.12)	1.13 (0.57–2.22) ^b^
20–29	23.7	17.2	24.4	1.37 (0.45–4.13)	1.25 (0.39–4.01) ^b^	23.2	23.8	1.06 (0.51–2.19)	1.20 (0.56–2.60) ^b^
30+	15.9	8.6	16.6	Reference	Reference	14.7	16.1	Reference	Reference
In Quartiles (ng/mL)									
Q1: <15	40.9	53.4	39.6	2.61 (0.98–6.91)	2.89 (1.02–8.18) ^b^	36.8	41.6	0.97 (0.49–1.88)	0.94 (0.45–1.92) ^b^
Q2: 15–21	25.2	25.9	25.1	1.99 (0.70–5.67)	1.89 (0.62–5.78) ^b^	29.6	24.4	1.32 (0.65–2.65)	1.48 (0.69–3.14) ^b^
Q3: 22–29	18.0	12.1	18.6	1.25 (0.38–4.08)	1.24 (0.35–4.30) ^b^	18.9	17.8	1.16 (0.54–2.47)	1.25 (0.56–2.77) ^b^
Q4: 30+	15.9	8.6	16.7	Reference	Reference	14.7	16.2	Reference	Reference
									

^a^ Adjusted for maternal age, parity, BMI before pregnancy, medical or obstetrical history, smoking, ethnicity, skin color and season at blood draw. ^b^ Adjusted for maternal age, parity, BMI before pregnancy, medical or obstetrical history, smoking, ethnicity and season at blood draw.

## Supplementary Materials

The following are available online at https://www.mdpi.com/2072-6643/11/12/3042/s1, Table S1: Association between vitamin D status in the first trimester of pregnancy and gestational age and birthweight as continuous variables in the overall sample and by skin color.
