# Supplementary material for: First Trimester Maternal Vitamin D Status and Risks of Preterm Birth and Small-For-Gestational Age
Source: Nutrients. 2019 Dec 13;11(12):3042. doi: 10.3390/nu11123042 (PMC6950733; doi:10.3390/nu11123042)
Supplement: Supplementary file 1 [file nutrients-11-03042-s001.pdf]

Table S1. Association between vitamin D status in the first trimester of pregnancy and gestational age and birthweight as continuous variables in the overall sample and by skin color.

25-OHD concentrations in the 1st trimester of pregnancy	Total	Gestational age at birth		Birthweight	
		Crude β (CI 95%)	Adjusted β (CI 95%)	Crude β (CI 95%)	Adjusted β (CI 95%)
**Overall sample** n(%)		189 (6.7)	2624 (93.3)	336 (11.9)	2477 (88.1)
Level cut-off (ng/mL)					
<20	45.1	-0.2 (-0.4, -0.1)	-0.2 (-0.3, 0.02)[a]	-6.5 (-58.0, 45.0)	-9.7 (-63.7, 44.3)[a]
20-29	30.9	0.002 (-0.2, 0.2)	-0.02 (-0.2, 0.2)[a]	33.3 (-22.2, 88.7)	17.9 (-36.7, 72.4)[a]
30+	24.1	Reference	Reference	Reference	Reference
In quartiles (ng/mL)					
Q1: <15	27.0	-0.3 (-0.5, -0.1)	-0.2 (-0.4, -0.001)[a]	-3.7 (-60.9, 53.4)	-5.8 (-66.5, 54.9)[a]
Q2: 15-21	24.1	-0.1 (-0.3, 0.1)	-0.1 (-0.3, 0.07)[a]	-5.8 (-64.6, 52.9)	-17.0 (-76.2, 42.3)[a]
Q3: 22-29	24.7	0.05 (-0.1, 0.2)	0.02 (-0.2, 0.2)[a]	39.3 (-19.0, 97.8)	26.9 (-30.3, 84.2)[a]
Q4: 30+	24.1	Reference	Reference	Reference	Reference
**Light skin (type I to IV)** n(%)		131 (6.0)	2066 (94.0)	241 (11.0)	1956 (89.0)
Level cut-off (ng/mL)					
<20	40.8	-0.1 (-0.3, 0.1)	-0.07 (-0.3, 0.1)[b]	28.0 (-26.7, 82.7)	10.1 (-47.7, 68.0)[b]
20-29	32.9	-0.006 (-0.2, 0.2)	-0.03 (-0.2, 0.2)[b]	42.2 (-15.1, 99.5)	28.8 (-27.9, 85.4)[b]
30+	26.3	Reference	Reference	Reference	Reference
In quartiles (ng/mL)					
Q1: <15	23.2	-0.2 (-0.4, 0.1)	-0.08 (-0.3, 0.1)[b]	33.2 (-29.2, 95.6)	16.2 (-50.8, 83.2)[b]
Q2: 15-21	23.8	-0.09 (-0.3, 0.1)	-0.1 (-0.3, 0.1)[b]	19.0 (-42.9, 80.9)	-1.0 (-63.7, 61.6)[b]
Q3: 22-29	26.6	0.04 (-0.2, 0.2)	0.02 (-0.2, 0.2)[b]	49.0 (-11.2, 109.2)	37.7 (-21.7, 97.1)[b]
Q4: 30+	26.3	Reference	Reference	Reference	Reference
**Dark skin (type V to VI)** n(%)		58 (9.4)	558 (90.6)	95 (15.4)	521 (84.6)
Level cut-off (ng/mL)					
<20	60.4	-0.4 (-0.9, 0.1)	-0.4 (-0.9, 0.1)[b]	-83.8 (-226.0, 58.5)	-60.8 (-202.0, 80.4)[b]
20-29	23.7	0.07 (-0.5, 0.7)	0.09 (-0.5, 0.7)[b]	-9.3 (-172.9, 154.3)	-9.0 (-168.8, 150.8)[b]
30+	15.9	Reference	Reference	Reference	Reference
In quartiles (ng/mL)					
Q1: <15	40.9	-0.5 (-1.0, 0.06)	-0.4 (-1.0, 0.1)[b]	-71.8 (-221.1, 77.5)	-51.9 (-200.2, 96.4)[b]
Q2: 15-21	25.2	-0.2 (-0.8, 0.4)	-0.1 (-0.7, 0.5)[b]	-85.6 (-247.5, 76.3)	-61.9 (-222.4, 98.7)[b]
Q3: 22-29	18.0	0.1 (-0.5, 0.7)	0.08 (-0.5, 0.7)[b]	-10.5 (-184.4, 163.3)	-10.8 (-179.9, 158.2)[b]
Q4: 30+	15.9	Reference	Reference	Reference	Reference

a Adjusted on maternal age, parity, BMI before pregnancy, medical or obstetrical history, smoking, ethnicity, skin color and season at blood draw

b Adjusted on maternal age, parity, BMI before pregnancy, medical or obstetrical history, smoking, ethnicity and season at blood draw
